# The Burden of NAFLD and Its Characteristics in a Nationwide Population with Type 2 Diabetes

**DOI:** 10.1155/2016/2931985

**Published:** 2016-03-30

**Authors:** Gabriele Forlani, Carlo Giorda, Roberta Manti, Natalia Mazzella, Salvatore De Cosmo, Maria Chiara Rossi, Antonio Nicolucci, Paolo Di Bartolo, Antonio Ceriello, Pietro Guida, AMD-Annals Study Group

**Affiliations:** ^1^Unit of Metabolic Diseases and Clinical Dietetics, “Alma Mater Studiorum” University of Bologna, 40138 Bologna, Italy; ^2^Diabetes and Metabolism Unit, ASL Turin 5, 10023 Chieri, Italy; ^3^Unit of Internal Medicine, IRCCS Casa Sollievo della Sofferenza, 71013 San Giovanni Rotondo, Italy; ^4^Center for Outcomes Research and Clinical Epidemiology (CORE), 65124 Pescara, Italy; ^5^Diabetes Unit, AUSL Romagna, 48121 Ravenna, Italy; ^6^Biomedical Research Institute August Pi Sunyer (IDIBAPS) and Center for Biomedical Research in Diabetes and Associated Metabolic Disorders (CIBERDEM), 08036 Barcelona, Spain; ^7^AMD (Italian Association of Clinical Diabetologists), 00192 Rome, Italy

## Abstract

*Objective*. We studied the prevalence of nonalcoholic fatty liver disease (NAFLD) and its clinical correlates in a population of patients with type 2 diabetes mellitus (T2DM).* Methods*. Clinical data of 94,577 T2DM patients were retrieved from 160 diabetes clinics in Italy in a standardized format and centrally analyzed anonymously. After exclusion of 5967 cases (high or uncertain alcohol intake), in 38,880 the Fatty Liver Index (FLI) was used as a proxy for the diagnosis of NAFLD. Factors associated with FLI assessed NAFLD (FLI-NAFLD) were evaluated through multivariate analysis.* Results*. FLI-NAFLD was present in 59.6% of patients. Compared to non-NAFLD, FLI-NAFLD was associated with impairment in renal function, higher albumin excretion, HbA1c and blood pressure, lower HDL cholesterol, and poorer quality of care. ALT was within normal limits in 73.6% of FLI-NAFLD patients (45.6% if the updated reference values were used). The prevalence of FLI-NAFLD did not differ if the whole sample (94,577 cases) was examined, irrespective of alcohol intake.* Conclusions*. FLI-NAFLD was present in the majority of T2DM patients of our sample and metabolic derangement, not alcohol consumption, was mainly associated with the disease. FLI-NAFLD patients have a worse metabolic profile. ALT levels are not predictive of NAFLD.

## 1. Introduction

Both nonalcoholic fatty liver disease (NAFLD) and Type 2 diabetes mellitus (T2DM) are highly prevalent in the community and are frequently associated with each other [1], as part of the metabolic syndrome [2]. The link between the two diseases is insulin-resistance and compensatory hyperinsulinemia progressing to *β*-cell dysfunction in T2DM or to defective lipid metabolism and hepatic triglyceride accumulation in NAFLD. This also explains why NAFLD is very common in T2DM (around 70% using ultrasound techniques [1]) and why NAFLD patients are at high risk of T2DM [3]. Notably, in subjects with T2DM hepatic fat accumulation is more likely to progress to nonalcoholic steatohepatitis (NASH) and fibrosis than in non-T2DM cases [4, 5] and ultimately to bridging fibrosis/cirrhosis or to hepatocellular carcinoma (HCC) [6]. Finally, whereas NAFLD per se [7] and T2DM increase the risk of cardiovascular events and Chronic Kidney Disease [8], their combination accelerates the progression of macro- and microvascular complications [9], independent of which disease comes first.

Given the burden of T2DM and NAFLD in the community and the gloomy perspectives for the future considering the high prevalence of obesity, it would be important to increase the low awareness of this ominous association between the general practitioners and the specialists caring for hospital patients [10], who frequently miss to diagnose NAFLD. Normal liver enzymes do not exclude NAFLD [11] and ultrasonography of the liver is needed [12]. Screening with routine noninvasive scores might help diagnose NAFLD, select patients for ultrasonography, and plan the most appropriate glucose-lowering therapy to prevent both hepatic and systemic complications [13]. We used the Fatty Liver Index (FLI) [14] as a proxy of diagnosis of NAFLD to investigate the prevalence of NAFLD in a large population of T2DM subjects without at-risk alcohol intake and its correlations with cardiovascular risk factors and renal dysfunction markers. FLI is based on simple anthropometric data and routine biochemistry and proved effective in identifying subjects with fatty liver in the community [15] and in the insulin-resistance setting [16], as well as identifying cases at risk of all-cause, cardiovascular, and liver-related outcomes [17]. For our purpose we used the database of the Italian Association of Clinical Diabetologists (Associazione Medici Diabetologi (AMD)) Annals [18, 19].

## 2. Methods

This is a cross-sectional study. The AMD (Associazione Medici Diabetologi) Annals initiative, a continuous quality improvement initiative, has been described in more detail elsewhere [18, 19]. Briefly, a set of indicators to be used for benchmarking activities is collected annually from participating Diabetes Centers in a standardized format (AMD data file) and centrally analyzed anonymously. Participation of Diabetes Centers in the AMD-Annals initiative is on voluntary basis. Quality indicators include process measures evaluating diagnostic, preventive, and therapeutic procedures and outcome indicators measuring favorable and unfavorable modifications in patient health status. Furthermore, the use of antidiabetic, antihypertensive, and lipid lowering drugs is evaluated. All centers share the same software for data extraction from electronic medical records. The entire project is conducted through a physician-led effort, without allocation of extra resources or financial incentives.

**Figure 1:**
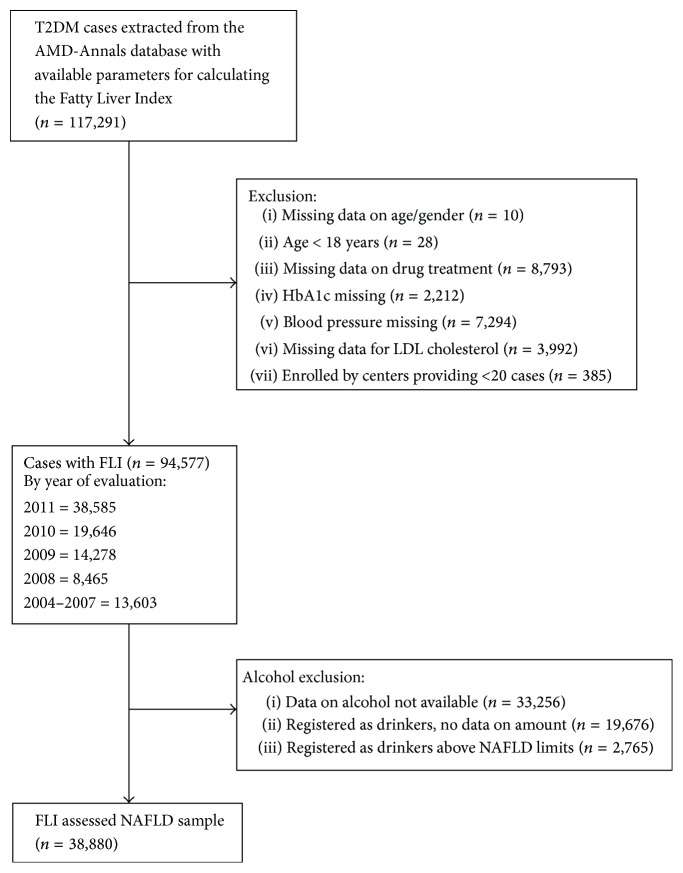
Flow chart of sample selection.

Clinical data extracted from electronic medical records used for everyday management of patients and collected during the period 2004–2011 in the database of AMD-Annals includes 942,784 T2DM patients from 302 Diabetes Centers. In this database we searched for patients with the complete dataset to calculate FLI (117,291 patients, from 160 Diabetes Units). After exclusion of 22,714 cases for incomplete laboratory values (Figure 1), 94,577 cases were left. Only 38,880 of them were definitely alcohol-free or with an alcohol intake below 30 g/day (males) or 20 g/day (females), as required by international associations for the diagnosis of NAFLD [20] and this was the sample used to calculate the prevalence of FLI-NAFLD. In cases of multiple records collected during the year for the same patient, the last available value was included. Data were generally captured in the same visit, but if data were not complete, missing data were captured in a period of 6 months. The FIB-4 score, a noninvasive test for hepatic fibrosis [21], was also used to evaluate the severity of NAFLD in the patients with FLI > 60 (probable NAFLD). Due to incomplete data presence in the database the FIB-4 was calculable in 15882/38880 patients (40.8%) with complete ALT, AST, and platelet count.

The FLI uses an algorithm based on body mass index (BMI (Kg/m^2^)), waist circumference (cm), triglycerides (mg/dL), and gamma-glutamyltransferase (GGT (IU/L)) as follows: FLI = 100 × exp⁡{0.953 × ln⁡(triglycerides) + 0.139 × BMI + 0.718 × ln⁡(GGT) + 0.053 × waist circumference − 15.745}/(1exp⁡{0.953 × ln⁡(triglycerides) + 0.139 × BMI + 0.718 × ln⁡(GGT) + 0.053 × waist circumference − 15.745}), where ln indicates the natural logarithm.

The FIB-4 algorithm is based on age (years), serum alanine aminotransferase (ALT (IU/L)), aspartate aminotransferase (AST (IU/L)), and platelet count (PLT count (10^9^/L)) according to the following formula: FIB-4 = (Age × AST)/(Platelet count × (square root of ALT)). A FIB-4 score > 3.25 has a positive predictive value to confirm the existence of a significant hepatic fibrosis.

Two cut-offs of ALT values were considered; the standard cut-off of the Italian laboratories of 41 U/L and 31 U/L for males and females, respectively, and the updated reference values of 31 IU/L and 19 IU/L [22]. LDL-C was estimated by the Friedwald equation. Albuminuria was defined as albumin excretion rate ≥20 mg/min, or albumin-creatinine ratio >2.5 (men) or >3.5 (women) mg/mmol, or microalbuminuria >30 mg/L. Glomerular filtration rate (GFR) was calculated with the Chronic Kidney Disease Epidemiology Collaboration formula (eGFR) [23].

Finally, in all cases the quality of care was estimated by the previously validated summary *Q* score, able to predict long-term outcomes in the AMD initiative dataset [24]. FLI, used as a proxy of the diagnosis of NAFLD [14] is based on simple parameters (BMI, waist circumference, triglycerides, and gamma-glutamyltransferase (GGT)), available in the database. FLI < 30 rules out and FLI ≥ 60 rules in hepatic steatosis as detected by ultrasonography.

### 2.1. Statistical Analysis

Continuous variables are expressed as mean standard deviation and discrete variables are expressed as percentage. We used multinomial logistic regression to estimate the relative risk ratios (RRRs) of FLI in the range 30–59 and FLI ≥ 60, compared with FLI < 30 (no steatosis, control). The multinomial logistic regression estimates the RRRs for observing a dependent variable with more than two categories as a function of independent covariates. Data were analyzed considering diabetes clinics as clusters of observations, so that possible differences in data across centers could be considered. Relative risk ratios were given with their 95% confidence intervals (CIs) considering patients with FLI < 30 as reference group. The analyses were made using STATA software, version 12 (StataCorp, College Station, Texas). *p* values of <0.05 were considered statistically significant.

## 3. Results

Patients were divided into 3 groups on the basis of FLI score: <30 (NAFLD-free), FLI 30–59 (possible NAFLD), and FLI ≥ 60 (probable NAFLD). Clinical features of patients according to Fatty Liver Index are reported in Table 1.

The group with FLI ≥ 60 (probable NAFLD) accounted for 59.6% of total patients, with the one with possible NALD (FLI 30–59) accounting for 25.2%. Patients with FLI ≥ 60 were more frequently males, younger, and with shorter duration of diabetes. As expected, the parameters included in the FLI algorithm (BMI, waist circumference, GGT, and triglycerides) increased from FLI < 30 to FLI value ≥ 60. Also the mean values of AST and ALT increased with increasing FLI categories, and HbA1c (mean value in the whole population 7.5%) showed a progressive deterioration. As to renal function, serum creatinine and albuminuria increased while eGFR decreased from FLI < 30 to FLI ≥ 60. Similarly, HDL cholesterol was significantly reduced with increasing FLI group, whereas less significant differences were observed in LDL cholesterol, also mediated by the prevalence of pharmacological therapy. Lipid lowering therapies were used in 49.6% versus 54.8% in FLI < 30 and FLI ≥ 60, respectively, and antihypertensive treatment was used in 57.7% and 75.9%. The increasing FLI score was also associated with an increased utilization of fibrates, biguanide, and thiazolidinediones, whereas insulin and sulphonamide utilization decreased and statins and aspirin showed minor differences. In spite of more frequent utilization of antihypertensive drugs, the mean values of blood pressure increased significantly with FLI values, and similarly plasma triglyceride levels increased in spite of a larger use of fibrates. The prevalence of out-of-range liver enzymes progressively increased with increasing FLI group. Considering the traditional reference values for ALT (≤31 UI/L for females and ≤41 for males), values within normal limits were present in 80% of patients, but the presence of abnormal values increased with FLI group (8.7, 12.8, and 26.4% in FLI < 30, 30–59, and ≥60 patients, resp.). When the updated reference values were considered (19 and 31 IU/L for females and males, resp. [21]), the prevalence of abnormal values increased several times to 32.0, 37.1, and 54.4% in the three groups with increasing FLI, respectively. After adjustment for gender, age, and duration of diabetes the relative risk ratios for FLI ≥ 60 increased with increasing AST/ALT levels, serum creatinine, albuminuria, HbA1c, and blood pressure values and with the presence of retinopathy. Also reduced eGFR and a worse quality of assistance (*Q* score) were associated with FLI score ≥ 60, while smoking did not differ between groups. AST > 38, ALT > 41, HDL cholesterol < 40 mg/dL (males) or < 50 (females), eGFR < 60 mL/min/1.73 m^2^, and blood pressure ≥ 140/85 mmHg increased the risk for FLI ≥ 60 (Table 2). In subjects with FLI ≥ 60 (probable NAFLD) the FIB-4 score resulted positive (>3.25) in 4.4%.

When the whole sample of 94,577 T2DM patients was examined, irrespective of alcohol consumption, no systematic differences were observed as compared to the alcohol-free sample (Table 3).

## 4. Discussion

In a large Italian population, our data confirm the high prevalence of NAFLD in T2DM. FLI positive subjects (FLI ≥ 60) represent about 60% of T2DM patients, and this figure probably underestimates the real prevalence of steatosis in the population since NAFLD might also be present in subjects with FLI 30–59, who are classified as uncertain by the score. We found a similar prevalence of FLI positive subjects in the non-alcohol consuming or light drinking NAFLD population and in the whole sample of patients irrespective of alcohol consumption. Therefore, in our community of subjects with T2DM metabolic factors are associated with fatty liver more than alcohol consumption. Several recent epidemiological studies support the importance of NAFLD in the population and in T2DM in particular. NAFLD is the most common cause of liver disease worldwide, the second leading etiology of liver disease among patients awaiting liver transplantation, and the most rapidly growing indication for liver transplantation in patients with hepatocellular carcinoma in the USA [25].

The relation between T2DM, NAFLD, and Cardiovascular Disease (CVD) is also well known [7, 8]. A recent systematic review points to an association between NAFLD and Chronic Kidney Disease (CKD) [8], also showing increased severity of NAFLD associated with an increased severity of CKD. Targher et al. reported an independent association of Chronic Kidney Disease (CKD) and proliferative/laser-treated retinopathy in T2DM [26] and this was the basis to support the concept that NAFLD might be included as a novel cardiometabolic risk factor for T2DM and its complications [7]. Our data confirm the association of NAFLD with CKD, with reduced eGFR and increased albumin excretion rate increasing the risk of NAFLD. The pathogenesis of this association is not completely explained. NAFLD and CKD share common risk factors and probably liver and kidney damage may be the consequence of obesity-driven mechanisms of disease as lipotoxicity, oxidative stress, proinflammatory state, and renin-angiotensin axis activation [27]. It is also possible that the steatotic and inflamed liver may promote liver injury through the production of inflammatory, profibrogenic, and antifibrinolytic molecules [28]. Contrary to previous data [29], in the AMD dataset kidney damage was not associated with a higher prevalence of retinopathy and the presence of retinopathy only slightly increased the risk of FLI assessed NAFLD, in keeping with a kidney damage not strictly related to diabetic microvascular disease. Diabetes accelerates disease progression and a recent analysis in an Italian diabetes population showed a 2.5-fold increased risk of dying from chronic liver disease, and particularly of non-alcohol- and non-virus-related causes, largely attributable to NAFLD [30]. In our patients with FLI assessed NAFLD the prevalence of advanced liver disease, defined by the FIB-4 score > 3.25, was 4.4%, so a relatively low proportion of NAFLD patients seem to have progressed to advanced liver disease. However from an epidemiological point of view if we consider the high prevalence of type 2 diabetes in general population and the very high prevalence of NAFLD in diabetic patients we are going to face a very large impact of NAFLD-related advanced liver disease on the health care system.

Diabetes specialists should diagnose NAFLD in their patients, but relying on normal liver enzymes to exclude NAFLD carries a high risk of underestimating the problem. In the present setting, mean ALT levels were higher in patients with FLI ≥ 60, but nearly half of patients with FLI ≥ 60 showed normal ALT values, also when grouped according to the updated reference cut-offs [22]. Noninvasive and inexpensive tests as FLI and FIB-4 may be very useful to diagnose the disease and to assess its degree of progression and finally to select patients for ultrasonography and additional work-up. In subjects diagnosed with NAFLD, any effort should be done to achieve glucose control by treatment(s) likely to reduce the burden of liver disease. Lifestyle intervention is the cornerstone of therapy for both diabetes and NAFLD, but pharmacological therapies for hyperglycemia may influence significantly the progression of NAFLD to NASH, cirrhosis, and liver cancer [6]. A few drugs for the treatment of hyperglycemia, hypertension, and dyslipidemia (metformin, thiazolidinediones, ACE inhibitors, ARBs, statins, and ezetimibe) might have protective activity on liver disease, while others are expected to worsen the disease [13]. In our dataset the pharmacological treatment of hyperglycemia seemed to be tailored to phenotypic characteristics of patients, and subjects with FLI ≥ 60, who had higher BMI and waist circumference, were more frequently treated with insulin sensitizers (metformin and particularly thiazolidinediones, that could improve the evolution of NAFLD) and less frequently with insulin and sulphonamides, as compared to patients with FLI < 30. In the same way ACE inhibitors and Angiotensin Receptor Blockers (ARBs), which seem to reduce insulin-resistance and give some advantages in NAFLD [1], were more frequently used in FLI ≥ 60. Low quality of care, assessed by *Q* score, is associated with an increased risk of FLI assessed NAFLD.

The strengths of our study are the large size of the sample, the unique data source, and the nonselection of patients, which makes the population representative of the general condition of NAFLD in T2DM outpatients of centers of diabetes care in Italy.

Our study has some limitations. Using formulas (FLI, FB4) as a proxy of diagnosis prevents the possibility of calculating the absolute prevalence of NAFLD (only a structured study directed to this aim with appropriate instruments as ultrasonography may be suitable for this purpose). Clinical data were extracted from electronic medical records used for everyday management out of any research design (only exams included in usual diagnostic protocols of participating centers were included), so only part of patients had the complete data-setting to calculate the FLI and could be included in the study; for this reason a selection of patients cannot be excluded. Nevertheless some data suggest that FLI availability did not select a sample with specific characteristics: in another analysis [31] performed on data extracted from the same database, with a different sample and different purposes, the metabolic and clinical characteristics of patients were identical to the patients included in our study.

Another limitation is the lack of possibility of ruling out chronic viral hepatitis. Hepatitis C Virus (HCV) may be responsible for the presence of hepatic steatosis, for elevation of AST/ALT, and for the progression of the disease. The positivity of the markers of viral hepatitis (HBV + HCV) in Italian diabetic patients has been reported in different centers between 5.2 and 10.8% [32], so the prevalence of NAFLD might be in part overestimated in our study and above all the rate of progression of the hepatic disease might be significantly linked to the presence of viral infections.

In conclusion we could confirm on a very wide population that NAFLD is largely present in type 2 diabetes and correlates with a worse metabolic profile and with organ damage. NAFLD must be searched for and regarded with special attention in type 2 diabetic patients. When NAFLD is present in type 2 diabetes a tailored approach to therapy is mandatory to prevent the progression of comorbidities and complications of diabetes (hepatic, renal, and cardiovascular).

## Figures and Tables

**Table 1:** Clinical features of patients according to Fatty Liver Index.

	All	Fatty Liver Index			*p* ^1^	*p* ^2^	*p* ^3^
		<30	30–59	≥60			
	*n* = 38880	*n* = 5882 (15.1%)	*n* = 9804 (25.2%)	*n* = 23194 (59.6%)			
Male gender	47.6%	41.0%	48.3%	49.0%	*δ*	*δ*	—
Age (years)	65 ± 12	66 ± 13	67 ± 11	64 ± 11	*α*	*δ*	*δ*
Known duration of diabetes (years)	9 ± 9	10 ± 10	9 ± 9	8 ± 9	*δ*	*δ*	*δ*
BMI (body mass index) (Kg/m^2^)	30 ± 6	24 ± 2	27 ± 3	33 ± 5	*δ*	*δ*	*δ*
Waist circumference (cm)	103 ± 13	87 ± 7	96 ± 6	110 ± 11	*δ*	*δ*	*δ*
GGT (UI/L)	39 ± 45	19 ± 14	28 ± 26	48 ± 52	*δ*	*δ*	*δ*
Triglycerides (mg/dL)	145 ± 99	88 ± 35	117 ± 50	172 ± 115	*δ*	*δ*	*δ*
Fatty Liver Index	64 ± 27	18 ± 8	46 ± 9	84 ± 12			
AST (UI/L)	24 ± 17	21 ± 12	23 ± 17	26 ± 19	*δ*	*δ*	*δ*
AST > 38 UI/L	8.4%	3.6%	5.3%	10.9%	*δ*	*δ*	*δ*
ALT (UI/L)	29 ± 23	22 ± 14	25 ± 21	32 ± 26	*δ*	*δ*	*δ*
ALT > 41 UI/L if male or >31 UI/L if female	20.3%	8.7%	12.8%	26.4%	*δ*	*δ*	*δ*
ALT > 30 UI/L if male or >9 UI/L if female	46.7%	32.0%	37.1%	54.4%	*δ*	*δ*	*δ*
Serum creatinine (mg/dL)	0.95 ± 0.51	0.89 ± 0.50	0.94 ± 0.51	0.97 ± 0.50	*δ*	*δ*	*δ*
eGFR (mL/min/1.73 m^2^)	79 ± 22	81 ± 20	78 ± 21	78 ± 22	*δ*	*δ*	—
eGFR < 60 mL/min/1.73 m^2^	19.7%	15.0%	19%	21.2%	*δ*	*δ*	*δ*
Albuminuria	26.8%	20.2%	23.4%	30.0%	*β*	*δ*	*δ*
HbA1c (% and mmol/mol)	7.5 (58) ± 1.6	7.3 (56) ± 1.6	7.3 (56) ± 1.5	7.6 (60) ± 1.7	—	*δ*	*δ*
HbA1c ≥ 7% (53 mmol/mol)	55.7%	49.0%	50.1%	59.8%	—	*δ*	*δ*
Total cholesterol (mg/dL)	184 ± 42	178 ± 38	180 ± 40	188 ± 43	*β*	*δ*	*δ*
HDL-C (mg/dL)	49 ± 14	58 ± 16	51 ± 14	46 ± 13	*δ*	*δ*	*δ*
HDL-C < 40 if male or <50 mg/dL if female	40.4%	21.4%	32.6%	48.5%	*δ*	*δ*	*δ*
LDL-C (mg/dL)	107 ± 36	103 ± 33	106 ± 34	108 ± 37	*δ*	*δ*	*δ*
LDL-C ≥ 100 mg/dL	54.7%	50.1%	53.6%	56.3%	*δ*	*δ*	*δ*
Systolic blood pressure (mmHg)	138 ± 19	134 ± 20	137 ± 19	139 ± 19	*δ*	*δ*	*δ*
Diastolic blood pressure (mmHg)	79 ± 10	76 ± 9	78 ± 10	80 ± 10	*δ*	*δ*	*δ*
Blood pressure ≥ 140/85 mmHg	55.6%	46.2%	53.1%	59.1%	*δ*	*δ*	*δ*
Pulse pressure (mmHg)	59 ± 16	58 ± 17	59 ± 17	59 ± 16	*δ*	—	*β*
*Q* score	27 ± 9	29 ± 8	28 ± 9	26 ± 9	*δ*	*δ*	*δ*
Retinopathy	12.0%	12.5%	12.1%	11.8%	—	—	—
Smokers	16.4%	15.6%	16.0%	16.9%	—	*α*	*β*
FIB-4 > 3.25 (%)				4.4%			
Lipid lowering treatment	54.2%	49.6%	55.5%	54.8%	*δ*	*δ*	—
Treatment with statins	49.1%	47.6%	51.6%	48.4%	*δ*	—	*δ*
Treatment with fibrates	3.4%	1.3%	2.4%	4.3%	*δ*	*δ*	*δ*
Antihypertensive treatment	71.6%	57.7%	69.7%	75.9%	*δ*	*δ*	*δ*
Treatment with ACE-Is/ARBs	59.8%	47.0%	57.3%	64.1%	*δ*	*δ*	*δ*
Aspirin	34.4%	31.8%	35.6%	34.6%	*δ*	*β*	—
Diabetes treatment:							
(i) Diet		8.2%	7.0%	5.9%	*α*	*δ*	*δ*
(ii) Insulin and metformin or sulfonamides	12.9%	10.4%	11.2%	14.2%	—	*δ*	*δ*
(iii) Metformin and sulfonamides	31.7%	28.9%	32.8%	31.9%	*δ*	*δ*	—
(iv) Metformin	25.1%	20.4%	24.1%	26.6%	*δ*	*δ*	*δ*
(v) Sulfonamides	8.0%	11.9%	9.3%	6.4%	*δ*	*δ*	*δ*
(vi) Other drugs	1.3%	1.4%	1.4%	1.2%	—	—	—
(vii) Thiazolidinediones	3.5%	3.0%	3.4%	3.6%	—	—	—
(viii) Dipeptidyl peptidase 4 inhibitors	2.2%	2.2%	2.3%	2.2%	—	—	—

Mean ± standard deviation or percentage of patients. ACE-Is, angiotensin converting enzyme-inhibitors; ARBs, Angiotensin Receptor Blockers; ALT, alanine transaminase; AST, aspartate aminotransferase; BMI, body mass index; eGFR, estimated glomerular filtration rate; GGT, gamma-glutamyltransferase; HDL-C, high-density lipoprotein cholesterol; LDL-C, low-density lipoprotein cholesterol. Missing data: AST in 1187 (1.3%), ALT in 1266 (1.3%), serum creatinine and eGFR in 3280 (3.5%), albuminuria in 15901 (16.8%), total cholesterol in 92 (0.1%), HDL-C (mg/dL) in 168 (0.2%), and smoking status in 101 (0.1%). The FIB-4 was calculable in 15882 (40.8%) patients with complete ALT, AST, and platelet count. The level of statistical significance for differences between groups is indicated by *p* ^1^ for FLI 30–59 versus FLI < 30, *p* ^2^ for FLI ≥ 60 versus FLI < 30, and *p* ^3^ for FLI ≥ 60 versus FLI 30–59.

*α*: *p* < 0.05, *β*: *p* < 0.01, *δ*: *p* < 0.001, and —: n.s.

**Table 2:** Relative risk ratios adjusted for gender, age, and duration of diabetes.

	RRR for FLI 30–59%	*p* ^1^	RRR for FLI ≥ 60%	*p* ^2^	*p* ^3^
AST (by 10 UI/L)	1.13 (1.07–1.20)	*δ*	1.29 (1.20–1.38)	*δ*	*δ*
AST > 38 UI/L	1.48 (1.22–1.79)	*δ*	3.06 (2.51–3.74)	*δ*	*δ*
ALT (by 10 UI/L)	1.27 (1.21–1.33)	*δ*	1.50 (1.40–1.62)	*δ*	*δ*
ALT > 41 UI/L	1.89 (1.65–2.15)	*δ*	4.54 (3.85–5.36)	*δ*	*δ*
ALT > 41 UI/L if male or >31 UI/L if female	1.63 (1.47–1.80)	*δ*	3.70 (3.24–4.23)	*δ*	*δ*
ALT > 30 UI/L if male or >19 UI/L if female	1.40 (1.30–1.51)	*δ*	2.78 (2.49–3.10)	*δ*	*δ*
Serum creatinine (by 1 mg/dL)	1.47 (1.23–1.77)	*δ*	1.93 (1.59–2.34)	*δ*	*δ*
eGFR (by 10 mL/min/1.73 m^2^)	0.92 (0.90–0.93)	*δ*	0.82 (0.81–0.84)	*δ*	*δ*
eGFR < 60 mL/min/1.73 m^2^	1.36 (1.26–1.48)	*δ*	2.11 (1.95–2.27)	*δ*	*δ*
Albuminuria	1.17 (1.03–1.32)	*α*	1.72 (1.43–2.07)	*δ*	*δ*
HbA1c (by 1%)	1.01 (0.98–1.04)	—	1.18 (1.11–1.25)	*δ*	*δ*
HbA1c ≥ 7% (53 mmol/mol)	1.10 (1.02–1.18)	*β*	1.71 (1.56–1.89)	*δ*	*δ*
Total cholesterol (by 20 mg/dL)	1.04 (1.02–1.06)	*δ*	1.13 (1.11–1.14)	*δ*	*δ*
HDL-C (by 10 mg/dL)	0.78 (0.76–0.80)	*δ*	0.57 (0.55–0.60)	*δ*	*δ*
HDL-C < 40 if male or <50 mg/dL if female	1.86 (1.73–2.00)	*δ*	3.53 (3.26–3.82)	*δ*	*δ*
LDL-C (by 20 mg/dL)	1.05 (1.03–1.07)	*δ*	1.08 (1.06–1.10)	*δ*	*δ*
LDL-C ≥ 100 mg/dL	1.17 (1.08–1.27)	*δ*	1.23 (1.14–1.34)	*δ*	*β*
Systolic blood pressure (by 20 mmHg)	1.18 (1.13–1.22)	*δ*	1.36 (1.28–1.44)	*δ*	*δ*
Diastolic blood pressure (by 10 mmHg)	1.20 (1.15–1.25)	*δ*	1.48 (1.40–1.56)	*δ*	*δ*
Systolic/diastolic blood pressure ≥140/85 mmHg	1.32 (1.24–1.40)	*δ*	1.78 (1.66–1.91)	*δ*	*δ*
Pulse pressure (by 10 mmHg)	1.04 (1.02–1.07)	*δ*	1.07 (1.04–1.10)	*δ*	*β*
*Q* score (by 10)	0.86 (0.83–0.90)	*δ*	0.67 (0.64–0.70)	*δ*	*δ*
Retinopathy	1.04 (0.94–1.15)	—	1.15 (1.05–1.26)	*β*	*α*
Smokers	1.00 (0.92–1.09)	—	0.92 (0.85–1.00)	—	*β*

Relative risk ratios (RRRs) with 95% confidence interval. The *p* values are referring to relative risk ratios for FLI 30–59 (*p* ^1^) or FLI ≥ 60 (*p* ^2^ and *p* ^3^) at multinomial logistic regression analysis correcting for gender, age, and duration of diabetes with patients with FLI < 30 (*p* ^1^ and *p* ^2^) and FLI 30–59 (*p* ^3^) as reference category.

*α*: *p* < 0.05, *β*: *p* < 0.01, *δ*: *p* < 0.001, and **—**: n.s.

**Table 3:** Relative risk ratios adjusted for gender, age, and duration of diabetes for the overall population.

	All	Fatty Liver Index			RRR for FLI 30–59%	*p* ^1^	RRR for FLI ≥ 60%	*p* ^2^	*p* ^3^
		<30	30–59	≥60					
	*n* = 94577	*n* = 13427	*n* = 24246	*n* = 56904					
AST (UI/L)	24 ± 16	21 ± 12	22 ± 14	26 ± 18	1.10 (1.06–1.15)	*δ*	1.35 (1.28–1.42)	*δ*	*δ*
AST > 38 UI/L	8.3%	3.2%	4.5%	11.2%	1.42 (1.24–1.61)	*δ*	3.53 (3.04–4.09)	*δ*	*δ*
ALT (UI/L)	28 ± 22	21 ± 14	24 ± 18	32 ± 25	1.26 (1.21–1.31)	*δ*	1.56 (1.48–1.66)	*δ*	*δ*
ALT > 41 UI/L (M) or >31 UI/L (F)	19.1%	7.8%	11.4%	25.0%	1.62 (1.49–1.76)	*δ*	3.88 (3.44–4.36)	*δ*	*δ*
ALT > 30 UI/L (M) or >19 UI/L (F)	43.4%	28.7%	33.1%	51.3%	1.41 (1.33–1.49)	*δ*	2.96 (2.71–3.23)	*δ*	*δ*
Serum creatinine (mg/dL)	0.97 ± 0.51	0.90 ± 0.45	0.96 ± 0.49	0.99 ± 0.53	1.60 (1.40–1.84)	*δ*	2.16 (1.82–2.58)	*δ*	*δ*
eGFR (mL/min/1.73 m^2^)	78 ± 21	80 ± 20	78 ± 20	77 ± 22	0.91 (0.90–0.92)	*δ*	0.82 (0.80–0.83)	*δ*	*δ*
eGFR< 60 mL/min/1.73 m^2^	19.8%	15.1%	19.1%	21.3%	1.41 (1.33–1.48)	*δ*	2.15 (2.03–2.28)	*δ*	*δ*
Albuminuria	28.2%	21.4%	24.9%	31.2%	1.20 (1.09–1.31)	*δ*	1.72 (1.46–2.04)	*δ*	*δ*
HbA1c (% and mmol/mol)	7.5 (58) ± 1.6	7.2 (55) ± 1.5	7.3 (56) ± 1.5	7.6 (60) ± 1.7	1.04 (1.01–1.07)	*β*	1.21 (1.15–1.27)	*δ*	*δ*
HbA1c ≥ 7% (53 mmol/mol)	55.1%	48.2%	50.2%	58.8%	1.16 (1.10–1.23)	*δ*	1.77 (1.64–1.91)	*δ*	*δ*
Total cholesterol (mg/dL)	185 ± 41	178 ± 38	180 ± 39	188 ± 43	1.04 (1.03–1.05)	*δ*	1.13 (1.12–1.14)	*δ*	*δ*
HDL-C (mg/dL)	50 ± 14	58 ± 16	52 ± 14	47 ± 13	0.79 (0.77–0.80)	*δ*	0.60 (0.58–0.62)	*δ*	*δ*
HDL-C < 40 (male); <50 mg/dL (female)	36.6%	19.5%	29.5%	43.7%	1.84 (1.74–1.95)	*δ*	3.43 (3.19–3.68)	*δ*	*δ*
LDL-C (mg/dL)	107 ± 36	104 ± 33	106 ± 34	109 ± 37	1.05 (1.04–1.07)	*δ*	1.07 (1.06–1.09)	*δ*	*δ*
LDL-C ≥ 100 mg/dL	55.3%	51.3%	54.2%	56.8%	1.15 (1.09–1.21)	*δ*	1.20 (1.14–1.27)	*δ*	*β*
Systolic blood pressure (mmHg)	139 ± 19	135 ± 20	138 ± 19	140 ± 19	1.17 (1.14–1.20)	*δ*	1.37 (1.31–1.43)	*δ*	*δ*
Diastolic blood pressure (mmHg)	79 ± 10	76 ± 9	78 ± 10	80 ± 10	1.20 (1.17–1.24)	*δ*	1.47 (1.41–1.54)	*δ*	*δ*
Blood pressure ≥ 140/85 mmHg	58.0%	48.7%	55.4%	61.3%	1.31 (1.25–1.38)	*δ*	1.76 (1.67–1.87)	*δ*	*δ*
Pulse pressure (mmHg)	60 ± 17	59 ± 17	60 ± 17	60 ± 16	1.04 (1.02–1.05)	*δ*	1.08 (1.05–1.10)	*δ*	*δ*
*Q* score	26 ± 9	28 ± 8	27 ± 9	26 ± 9	0.85 (0.82–0.87)	*δ*	0.67 (0.64–0.69)	*δ*	*δ*
Retinopathy	11.8%	12.5%	12.3%	11.5%	1.08 (1.00–1.17)	—	1.16 (1.07–1.26)	*δ*	*α*
Smokers	18.1%	17.6%	17.6%	18.3%	0.97 (0.90–1.03)	—	0.87 (0.82–0.93)	*δ*	*δ*
Alcohol	45.2%	41.9%	46.8%	45.4%	1.05 (0.98–1.13)	—	1.00 (0.93–0.07)	—	*α*

Mean ± standard deviation or percentage of patients. Relative risk ratios (RRR) with 95% confidence interval. ALT, alanine transaminase; AST, aspartate aminotransferase; eGFR, estimated glomerular filtration rate; HDL-C, high-density lipoprotein cholesterol; and LDL-C, low-density lipoprotein cholesterol. Missing data: AST in 4151 (4.4%), ALT in 3587 (3.8%), serum creatinine and eGFR in 8017 (8.5%), albuminuria in 43247 (45.7%), total cholesterol (mg/dL) in 211 (0.2%), HDL-C (mg/dL) in 440 (0.5%), smoking status in 30189 (31.9%), and alcohol in 32742 (34.6%). The *p* values are referring to relative risk ratios for FLI 30–59 (*p* ^1^) or FLI ≥ 60 (*p* ^2^ and *p* ^3^) at multinomial logistic regression analysis correcting for gender, age, and duration of diabetes with patients with FLI < 30 (*p* ^1^ and *p* ^2^) and FLI 30–59 (*p* ^3^) as reference category. RRR for continuous variables by 10 units' increase in AST, ALT, eGFR, HDL-C, diastolic blood pressure, pulse pressure, and *Q* Score, by 20 units for total cholesterol, LDL-C, and systolic blood pressure, and by one unit otherwise.

*α*: *p* < 0.05, *β*: *p* < 0.01, *δ*: *p* < 0.001, and **—**: n.s.
